# Malignant melanotic schwannoma of the cervical spinal cord: a case report

**DOI:** 10.1186/s12883-024-03686-0

**Published:** 2024-05-30

**Authors:** Sihan Chen, Yiting Wei

**Affiliations:** 1 Department of Neurosurgery, Taikang Ningbo Hospital, Ningbo, China; 2 Department of Neurosurgery, The First Affiliated Hospital of Ningbo University, Ningbo, Zhejiang China

**Keywords:** Malignant melanotic schwannoma, Malignant tumors of the central nervous system, Malignancy, Spinal tumors

## Abstract

Spinal cord malignant melanotic schwannoma (MMNST) is a rare central nervous system tumor that originates from the spinal cord or spinal myelin sheath cells and can produce melanin. This type of tumor is usually highly aggressive and malignant, with a poor prognosis. The clinical manifestations of spinal cord MMNST are mainly pain, paresthesia, muscle weakness, muscle atrophy, etc., and symptoms of spinal cord compression, such as intestinal and bladder dysfunction, paraplegia, etc. Early detection of tumor lesions can facilitate tumor removal, improve patients’ quality of life, and prolong patients’ survival. In this case report, a 27-year-old young woman was diagnosed with MMNST of the cervical spinal cord due to weakness of her limbs in our hospital, and underwent surgical resection. The patient’s limbs returned to normal after surgery. It is worth mentioning that the patient visited our hospital 7 months ago for “right upper limb pain for 3 days” and was diagnosed with a cervical spine space-occupying lesion at the same position this time, but the pathology report was “hemosiderosis”. This case report aims to raise awareness of the problem of spinal cord MMNST and contribute to greater knowledge of this rare tumor.

## Introduction

MMNST is a rare subtype of a schwannoma that can produce a melanin. It usually occurs in the cervical and thoracic segments of the spinal nerves, but can also occur in the head, face, orbit, oral cavity, and other parts of the spinal nerves. The tumor is highly malignant and often metastasizes and recurs, with a poor prognosis [1]. The etiology of the MMNST is unknown. Some studies have speculated that it may be related to genetic factors, ionizing radiation, or malignant transformation of a neurofibromas, but there is no clear evidence [2–4]. The MMNST may compress or invade the affected nerve, causing sensory or motor impairment in a corresponding area. For example, radicular pain, numbness, weakness, hemiplegia, etc. If the cranial nerves are involved, there may also be tinnitus, hearing loss, facial numbness or a pain, facial paralysis, dysphagia, etc. The most common sites of the metastasis are the lungs, followed by the bones, and can also be seen in the pleura, the retroperitoneum, etc. The metastasis may cause impairment of the function of a corresponding organ, such as dyspnea, cough, chest pain, bone pain, etc. The diagnosis of the MMNST mainly relies on imaging examinations and a pathological biopsy [3]. The treatment of the MMNST is mainly a surgical resection, and sometimes radiotherapy and chemotherapy are also needed. The survival period of the MMNST is very short, generally not more than one year. Early detection and a treatment may improve the quality of life and prolong the survival of patients. This paper is a case report of a patient with the MMNST, hoping to increase the clinical diagnosis rate of this disease and let patients get earlier and better a treatment [5].

## Case report

**Fig. 1:**
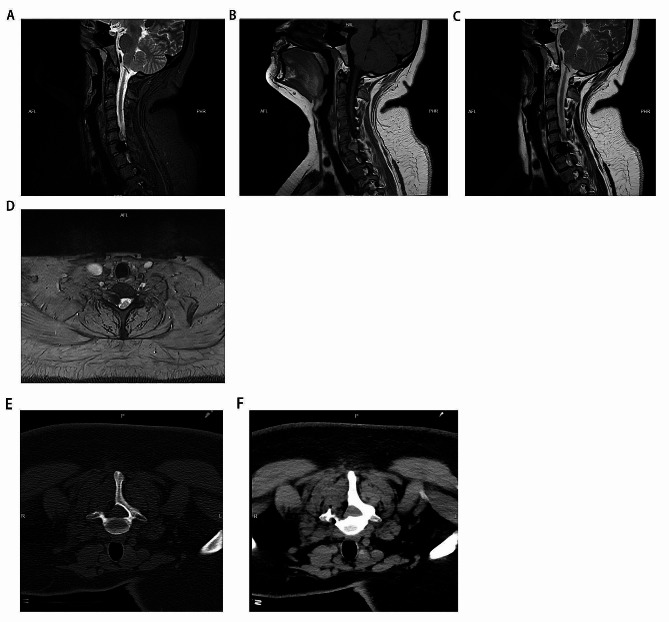
MRI of the cervical spine, showing a space-occupying lesion in the cervical spine; (**A**) MRI enhancement, cervical sagittal view; (**B**) T1 item, cervical sagittal view; (**C**) T2 cervical sagittal position; (**D**) T1, cross-section. (**E**) Shows the bone window after surgery; (**F**) Shows the tissue window after surgery

**Fig. 2:**
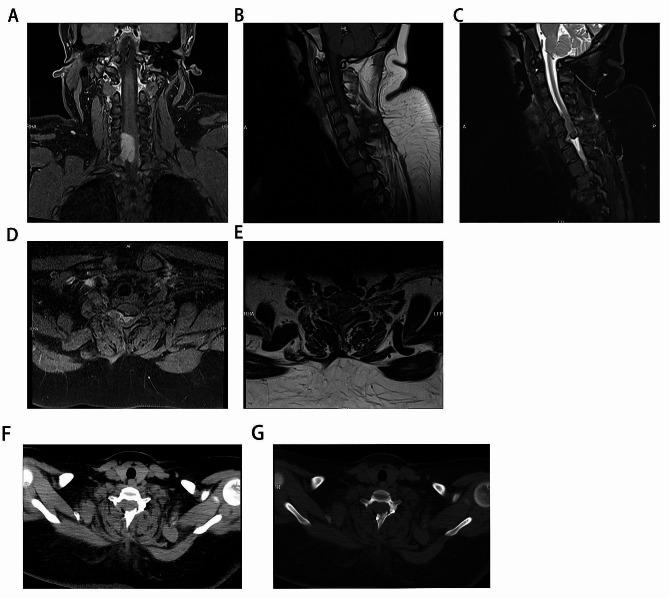
Pathology report. (**A**) Pathological microscopic image of the first operation; (**B**) Pathological microscopic image of the second operation

Patient information: A 27-year-old female who came to our hospital on March 11, 2023 with pain and numbness in her right hand. There was no loss of muscle strength in the limbs, and there was no autonomic dysfunction. MRI showed that C7-T1 extraspinal lesions in the neuraxial canal were more likely to have vascular malformations (Fig. 1). After discussion, under neurophysiological monitoring, microscopic neuraxial tumor resection was performed. Postoperative pathological findings showed that a large number of fibrovascular tissues were accompanied by hemorrhage, degeneration, and hemosiderin deposition. Considered vascular malformations, immunohistochemistry: Red-KI-67 (-), Red-Melan A (-), Red-Melanoma (-), Red-S-100 (-), CD31 (-), SMA (+), NF (+), GFAP (+) (Fig. 2).

**Fig. 3:**
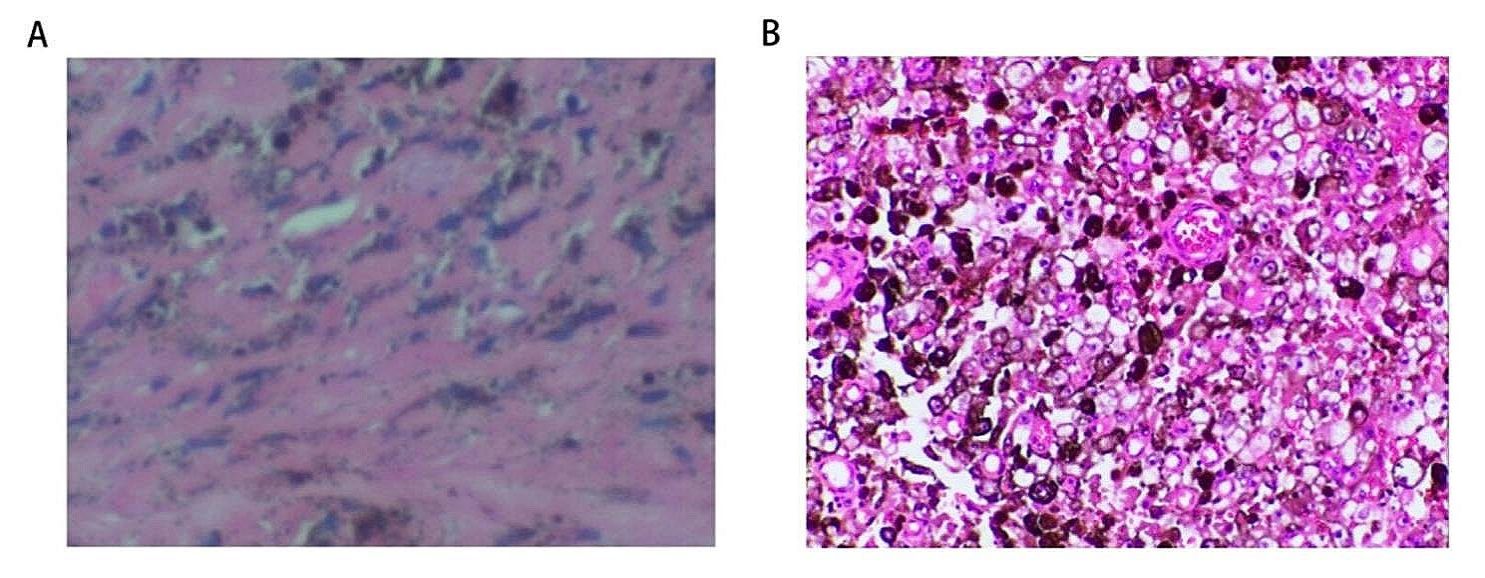
MRI of the cervical spine, showing a space-occupying lesion in the cervical spine. **A.** Enhancement item, coronal position; **B.** MRI enhancement, cervical sagittal view; **C.** T1 item, cervical sagittal view; **D.** T1 cross-section.; **E.** T2, cross-section. **F.** Shows the tissue window after surgery; **G.** Shows the bone window after surgery

**Fig. 4:**
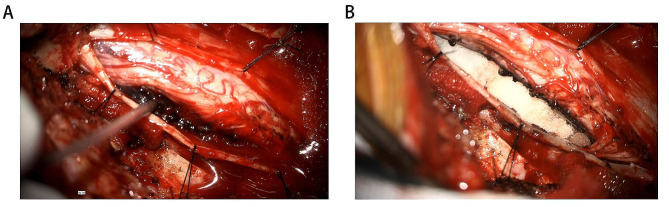
Microscopic lesions. (**A**) Before surgical resection; (**B**) After surgical resection

The patient developed paralysis of both lower limbs 7 months later and was re-admitted to our hospital. Physical examination: left lower limb muscle strength grade 1, right lower limb muscle strength grade 0, both upper limb muscle strength grade 3. No obvious superficial or deep sensory impairment, pathological signs suspiciously positive. MRI showed that C5-7 intraspinal canal extraspinal cord mass lesions, about 20 mm×18 mm×34 mm in size, slight compression of the cervical spinal cord, and no obvious enhancement on contrast scan. Schwannoma is a high likelihood (Fig. 3). After the contraindications to surgery were ruled out, under neurophysiological monitoring, microscopic neuraxial tumor resection was performed. Surgical situation: The tumor is located on the right ventral side of the spinal canal, which is black, medium in texture, and has a rich blood supply, eroding the surrounding tissues. A small amount of tumor tissue protrudes through the intervertebral foramen, and the spinal cord compresses to the left, which is obviously compressed and deformed. Under the microscope, the tumor is separated along the basilar and the spinal canal is smaller, and the tumor is removed at the same time as the basal separation, gradually reducing the tumor mass effect. Excision of tumors within the spinal cord. Then, using a peeler, carefully separate and scoop out a small amount of tumor from the foraminum. Delicately remove all tumor tissue visible to the naked eye. Dural tone is not high, and no active bleeding is seen after subdural irrigation. The dura mater is sutured with an artificial dural patch and reinforced with bioglue. The spinous processes and lamellar are reduced and fixed with titanium alloy connecting plates and homologous screws. After the instruments and gauze are counted correctly, the muscle layer, deep fascia, subcutaneous tissue, and skin are layered, intermittently, and tightly sutured. The operation was completed, the operation went smoothly, the intraoperative blood loss was about 200 ml, and there was no blood transfusion. Immunohistochemical results, MMNSST is considered (Fig. 4). Immunohistochemistry: SOX-10 (+), S-100 (+), Red-KI-67 (7%), Ki-67 (+), Melan-A (A103) (+), Melanoma (HMB45) (+), Red-Melan A (+), Red-Melanoma (+), CK (pan) (-), EMA (-), GFAP (-).

## Discussion

Neuraxial extramedullary tumors mainly include extramedullary intradural tumors and epidural tumors. Among them, extramedullary intradural tumors account for about 55% of neuraxial tumors. Schwannomas are mostly benign. MMNST is a rare neurogenic sarcoma that can produce melanin. It usually occurs in the cervical and thoracic spinal nerves, but can also occur in the head, face, orbit, oral cavity and other parts. It belongs to a highly malignant tumor, the recurrence rate of MMNST may be as high as 35% and the rate of distant metastasis as high as 44%, and has a poor prognosis. The cause of MMNST is unknown, and may be related to genetic factors, radiation exposure or malignant transformation of neurofibromas [6, 7]. Treatment of malignant melanotic schwannoma is mainly surgical, and in principle, radical resection is required. Incomplete resection often results in an increased rate of tumor recurrence. Patients should be followed up for a long time postoperatively, including screening for Carney syndrome, especially in younger patients [3]. The efficacy of chemoradiotherapy for this disease is uncertain, but there is some literature suggesting that stereotactic radiotherapy can be used to treat this disease, but more evidence-based evidence is lacking [8, 9].

In this case, the patient was admitted to the hospital on November 5, 2022 with pain and numbness in his right wrist. At that time, imaging and clinical diagnosis mainly considered spinal hemangioma, and although the tumor was completely removed, the patient had a recurrence of limb weakness and numbness after 7 months. It is worth mentioning that the first pathological report did not accurately identify MMNST, and related studies also proved that the disease is still difficult to identify in the field of pathology [10, 11]. This study focuses on MMNST to be misdiagnosed, and at the time of our first consultation, it was considered to be a vascular malformation, and black deposits were observed at the time of surgical resection, and it was considered to be a change in hemosiderin deposition at that time. Even the pathologic diagnosis suggests a benign lesion. However, after 7 months, the tumor recurred, and it was only then that we realized that the disease was not benign. This case report hopes to provide you with more information about MMNST, on the one hand, to reduce the misdiagnosis of the disease, to pay more attention to the complete removal of the lesion during surgery, and on the other hand, to provide a pathological tissue for those who need to be studied [9].

## Data Availability

Data is provided within the manuscript files.

## Abbreviations

MMNST: Malignant melanotic schwannoma
